# Cyan color-emitting nitrogen-functionalized carbon nanodots (NFCNDs) from *Indigofera tinctoria* and their catalytic reduction of organic dyes and fluorescent ink applications†

**DOI:** 10.1039/d1ra04351j

**Published:** 2021-08-16

**Authors:** Jothi Vinoth Kumar, Ganesan Kavitha, Rajaram Arulmozhi, Velusamy Arul, Natarajan Abirami

**Affiliations:** Department of Chemistry, College of Engineering and Technology, SRM Institute of Science and Technology Kattankulathur Tamil Nadu-603 203 India abiramin@srmist.edu.in; Department of Science and Humanities, Sri Venkateswaraa College of Technology Vadkkal, Sriperumbudur Chennai-602 105 Tamil Nadu India

## Abstract

The present study reports the synthesis of nitrogen-functionalized carbon nanodots (NFCNDs) by a low-cost hydrothermal method using the leaf extract of *Indigofera tinctoria* as a novel carbon precursor. The synthesized NFCNDs were characterized by diverse spectroscopic techniques. The optical properties of N-CNDs were analyzed by UV-visible and fluorescence spectroscopic studies. The quantum yield (QY) for the prepared NFCNDs was found to be 12.6%. The surface morphology, functional groups, and crystallinity of NFCNDs were evaluated by HR-TEM, FT-IR, XRD and Raman spectroscopic methods, respectively. The Raman results revealed the moderate graphite structure of NFCNDs, and the calculated *I*_D_/*I*_G_ value was 0.49. The spherical appearance of the synthesized NFCNDs was confirmed by HR-TEM, and the calculated size of the NFCNDs was 4 nm. The XRD and SAED pattern results gives an evidence for the amorphous nature of the prepared NFCNDs. The thermal stability of NFCNDs was studied by TGA analysis. The resulting NFCNDs acted as a green nanocatalyst and thus efficiently improved the reducing capability of sodium borohydride (NaBH_4_) in the catalytic reduction of methylene blue (MB) and methyl orange (MO) dyes. Furthermore, the bright cyan emission characteristics of synthesized NFCNDs were utilized as a labeling agent in anti-counterfeiting applications.

## Abstract 2

The present study reports the synthesis of nitrogen-functionalized carbon nanodots (NFCNDs) by a low-cost hydrothermal method using the leaf extract of *Indigofera tinctoria* as a novel carbon precursor.

## Graphical abstract

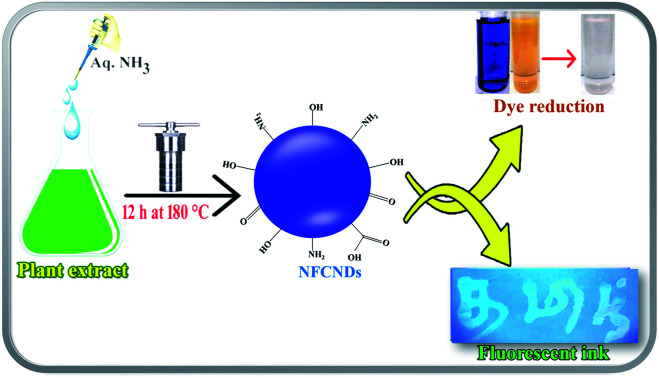

## 1 Introduction

Carbon-based nanomaterials such as carbon nanotubes,^1^ graphene oxide,^2^ fullerenes,^3^ and carbon nanodots^4,5^ have been playing vital roles in science and technology in the past two decades. Among these, carbon nanodots (CNDs) are fluorescent carbon nanomaterials that have been engaged at the forefront of recent research.^6^ Carbon nanodots (CNDs) are nanoparticles 1–10 nm in size, which were unexpectedly discovered by Anbu *et al.* during the purification of single-walled carbon nanotubes in 2004.^7^ The properties of the CNDs mainly depend on two factors: first, the choice of the carbon source (raw material), and second, the synthesis method. CNDs are quite divergent from other carbon-based nanomaterials due to their exclusive outstanding properties, such as high water dispersibility, strong chemical inertness, fluorescence, excellent photostability, less toxic nature, excellent bio-compatibility, environmental friendliness, low cost and flexibility in surface modification.^8,9^ CNDs have multipurpose applications in various fields, such as drug delivery, optoelectronic devices, sensing of metal ions, biological imaging, biomolecules, catalysis, solar cells, and the oxygen reduction reaction.^10–16^

CNDs have been synthesized by various top-down methods, including laser ablation, ultrasonic treatment, arc-discharge, and electrochemical and chemical oxidation, and bottom-up methods such as hydrothermal carbonization, microwave irradiation, pyrolysis, and plasma treatment.^17^ Among these synthesis methods, arc discharge and laser ablation processes need highly expensive instruments, whereas electrochemical oxidation and chemical oxidation need strong acids. The microwave irradiation method demonstrates a simple way to synthesize CDs within few minutes; this method can be restricted by uncontrollable reaction conditions. The hydrothermal method is simplistic, quick, and economical, has a simple experimental setup, is and eco-friendly when compared to other synthesis methods. Carbon dots with tunable degrees of carbonization usually consist of carbon (C), oxygen (O), and hydrogen (H) decorated with various functional groups on their surfaces. Using inexpensive and eco-friendly biomass sources such as *Phyllanthus emblica*, prickly pear cactus, rice bran, *Coccinia indica*, and sweet potatoes as carbon precursors to harvest CNDs has attracted great attention because of their green approach.^18–22^ The plant extracts containing citric acid, tartaric acid, and ascorbic acid act as efficient carbon precursors for the synthesis of highly fluorescent CNDs. The fluorescence properties of CNDs are influenced by solvents, size, pH, and dopants used during the synthesis. Recently, surface functionalization on CNDs was achieved by doping heteroatoms (such as boron, sulphur, nitrogen, and phosphorous). The functionalization of nitrogen tremendously increases the optical properties of CNDs when compared to undoped CNDs. At the same time, other important features of CNDs, such as the quantum yield (QY) and fluorescence lifetime, are significantly improved by the functionalization approach. Further, the heteroatom doping affects the internal sharing of electrons in CDs and thus can change the bandgap energy, which results in escalated fluorescence intensity of CNDs.^23,24^ These functionalized CNDs act as remarkable fluorescent probes in bio-imaging, catalysis, and chemo-sensing applications.^25–28^

In this work, *Indigofera tinctoria* (*I. tinctoria*), which belongs to the Fabaceae family, is used as a carbon precursor. This plant with medicinal values is found in various places in India. The phytochemical constituents present in the *I. tinctoria* leaf extracts are alkaloids, proteins, free amino acids, phenolic compounds, flavonoids, *etc.* The extracts are very useful for hair growth, epilepsy, chronic bronchitis, ulcers, skin diseases, and asthma, and are also used in cancer treatment.^29^ Due to the availability of rich bioactive components, *I. tinctoria* leaf extracts are widely used for many biological activities, such as antifungal, antimicrobial, antioxidant, anti-seborrhea, anti-psoriatic, anti-inflammatory, and anti-acne.^30,31^ Besides the above merits, *I. tinctoria* leaf extract was elected as a novel carbon precursor for the synthesis of CNDs.

**Fig. 1:**
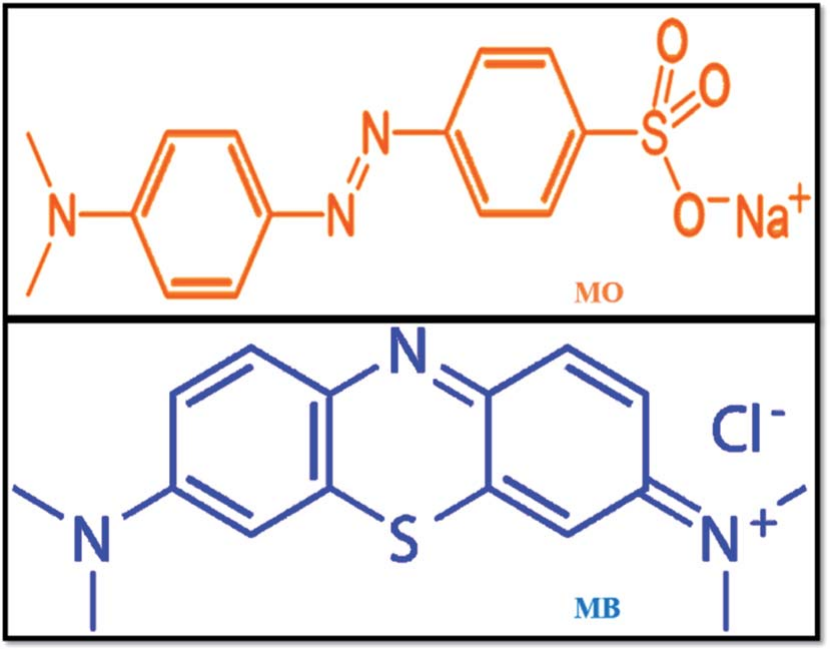
Structures of methyl orange and methylene blue.

With the rapid development of the dyeing industry, pharmaceutical industries, and printing technology, untreated sewage is being mixed into lakes and rivers every day, which is very harmful to humans and also to marine aquatic organisms. Hence, it is now a critical situation to treat organic dye-containing industrial wastewater using current technologies.^32^ Various methodologies have been adopted for the removal of harmful pollutants from waste effluents, such as adsorption,^33^ Fenton oxidation,^34^ photocatalytic reduction,^35^ and adsorbent membrane separation.^36^ Methyl orange (MO) is a sulphonated azo dye; due to its high toxicity, it causes harmful effects to human health and aquatic organisms.^37^ MO is widely used in textiles and in chemical laboratories as a pH indicator (p*K*_a_ = 3.5). Further, most industries use methylene blue (MB) dye, which is a highly carcinogenic thiazine dye, as a coloring agent. MB creates health problems, such as eye burns, skin irritation, cancer, and allergic dermatitis, for humans and animals. At present, it is very necessary to take action to remove these harmful pollutants from the environment using modern technologies. The chemical structures of MO and MB dyes are shown in Fig. 1.

Counterfeiting is a major problem owing to its wide-ranging impact on various companies, governments, and customers. Therefore, it is mandatory to produce anti-counterfeiting agents that ease the identification of fake currencies, including public documents and certificates.^38^ CNDs seem to be advantageous as anti-counterfeiting agents, as they possess good fluorescence properties, great quantum yields, low cytotoxicity, and abundant sources. To our knowledge, the anti-counterfeiting applications of NFCNDs obtained from *I. tinctoria* plant extract as a fluorescent material have not been reported yet.

In the present research contribution, the conversion of waste pollutant water to commercial material is reported *via* the green synthesis of blue-fluorescent nitrogen functionalized carbon nanodots (NFCNDs) using easily available *Indigofera tinctoria* (*I. tinctoria*) leaf extract as a carbon source with aq. ammonia as the nitrogen (N) source by a hydrothermal carbonization process. The prepared NFCNDs were carefully characterized by important spectroscopic tools. Furthermore, the catalytic ability of the NFCNDs was carefully examined in the reduction of MB and MO in the presence of NaBH_4_ (reducing agent). Also, owing to their highly fluorescent properties, the NFCNDs may be used as an anti-counterfeiting agent.

## 2 Materials and methods

### 2.1 Chemicals and reagents

The *Indigofera tinctoria* leaf was collected from Nathapattu Village, Cuddalore District of Tamil Nadu, India. Aqueous ammonia, methylene blue, methyl orange, sodium borohydride, and polyvinyl alcohol (PVA) were purchased from Avra Synthesis. All laboratory-grade chemicals and reagents were used exclusive of addistional purification. Double distilled water was used for solution preparation and other purposes during the entire study.

### 2.2 Extract preparation

Freshly collected *Indigofera tinctoria* plant leaf (50 g) was carefully washed with distilled water to remove impurities. The cleaned leaf was homogenically ground in an electric mixer by adding 100 mL of double-distilled water. The mixture was continuously heated at 30 °C, stirred for 1 h and then filtered, first by using Whatman filter paper and then by centrifugation at 10 000 rpm for 15 min; finally, the supernatant was collected and used as a carbon source for the synthesis of NFCNDs.

### 2.3 Synthesis of NFCNDs

For the green synthesis of NFCNDs, 49 mL of *Indigofera tinctoria* leaf extract was mixed with 1 M solution of aq. NH_3_ (1 mL), which was transferred to a 100 mL autoclave (Teflon-lined) and placed inside a hot air oven at 180 °C for 12 hours. After 12 h of the hydrothermal process, the autoclave was allowed to cool to room temperature; it was then carefully opened, and the formation of NFCNDs was visually confirmed by the observed color change from light green to dark brown. The obtained solution containing NFCNDs was filtered by Whatman 40 filter paper and further subjected to centrifugation at 10 000 rpm for 40 min to afford pure NFCNDs without large carbon residues or unreacted organic moieties. The obtained NFCNDs solution was further filtered by a 0.2 μm membrane to afford the pure NFCNDs, which were collected in an amber glass bottle and stored at 4 °C for further characterization and applications.

### 2.4 Instrumentation

The synthesized NFCNDs were examined and confirmed by various spectroscopic studies. The characteristics of the NFCNDs were analyzed by UV-visible spectra (Shimadzu UV-2600 spectrophotometer) and a HORIBA JOBIN YVON F-luromax-4 spectrofluorometer, respectively. The recorded absorption and emission data were plotted using “UVPROBE’’ software. The various functionalities present on the synthesized NFCNDs were explored by an Agilent Resolution FTIR spectrometer in the operating range between 400 and 4000 cm^−1^. A PAN analytical X'Pert power diffractometer was used to examine the amorphous nature of the NFCNDs using a Cu Kα radiation source with a range of 10 to 80° (2*θ*). The structural morphology of the NFCNDs was analyzed by a JOEL/JEM 2100 high resolution transmission electron microscope (HR-TEM) at an operating voltage of 200 kv using a carbon-coated Cu grid. The electronic states and elemental analyses of the NFCNDs were determined by recording XPS spectra using a Kratos Axis XPES spectrometer containing Al Kα X-rays as an electron beam source. Raman spectra were obtained using a Renishaw Raman scope at 633 nm (*λ*_ex_).

### 2.5 Quantum yield measurement

The quantum yield (QY) of the prepared NFCNDs was calculated using eqn (1). The QY was calculated using the reference of 0.1 M quinine sulfate (QY = 0.54%) dissolved in H_2_SO_4_ at 370 nm.^39^

1 *Q*_*x*_ = *Q*_std_(*I*_*x*_/*I*_std_)(*η*_*x*_^2^/*η*_std_^2^)(*A*_std_/*A*_*x*_)

where *Q*_*x*_ and *Q*_std_ are the quantum yields of NFCNDs and QS, while *I*_*x*_ and *I*_std_ are the fluorescence emission intensities, *A*_*x*_ and *A*_std_ are the optical densities, and *η*_*x*_^2^ and *η*_std_^2^ are the refractive indices of the reference and NFCNDs, respectively.

### 2.6 Evaluation of the photocatalytic reduction of NFCNDs

The reduction of MB and MO dyes using sodium borohydride (NaBH_4_) with the help of the prepared NFCNDs catalyst was performed under the irradiation of visible light produced by a 300 W xenon lamp. For conducting the photocatalytic reaction, 1 mL of freshly prepared ice-cold NaBH_4_ (0.05 M) was separately added to 14 mL (10 ppm) of newly prepared MB and MO dye solution, and 15 mL of DD water was also added to the mixture. To start the catalytic reduction reaction, the identical mixture of the initial reaction solution was taken, and 60 μL of catalyst (NFCNDs) was added to the MO and MB reaction mixtures. The solution was placed at 80 mm away from the visible light source (xenon lamp) and irradiated with slow stirring until dye decoloration occured. A 2.5 mL sample was taken at linear time intervals, and the reduction dynamics (absorption intensity) were observed using UV-vis spectroscopy (Shimadzu UV-2600).

### 2.7 Preparation of NFCNDs fluorescent ink

For the preparation of the anti-counterfeiting fluorescent ink, a 5 mL NFCNDs sample was dissolved in 10 mL of 5% polyvinyl alcohol under ultrasonic treatment for 10 min. This mixture was magnetically stirred for 2 h until a high-viscosity ink formed. Next, the prepared ink was transferred into an empty refill tube, and hand-writing or drawing of images on normal filter paper was performed; then, the ink was allowed to dry in ambient air. After 2 h, the dried paper was examined under UV light at 365 nm and the obtained visible images were captured using a camera.

## 3 Results and discussion

**Scheme 1:**
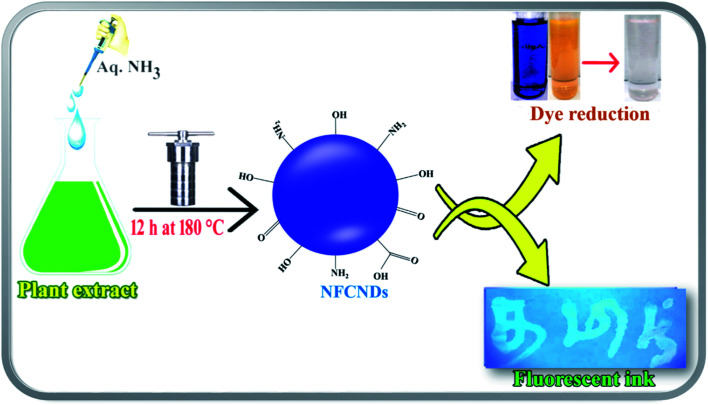
Schematic of the synthesis and application of NFCNDs.

The leaf extract of *I. tinctoria* and aq. NH_3_ were employed as the carbon and nitrogen source, respectively. The formation of NFCNDs was preliminarily confirmed thorough visual observation of the color change. The reaction time and temperature play crucial roles in the formation of highly fluorescent and stable NFCNDs with desired properties.^40^ The graphical representation of the hydrothermal synthesis of NFCNDs is given in Scheme 1.

### 3.1 UV-visible and fluorescence spectra of the synthesized NFCNDs

**Fig. 2:**
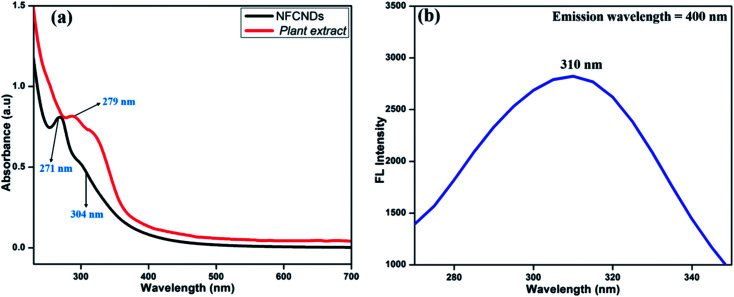
(a) UV-vis absorbance spectra of the plant extract and prepared NFCNDs; (b) fluorescence excitation spectrum of the prepared NFCNDs.

Fig. 2(a) displays the UV-vis spectrum of the carbon source (*I. tinctoria* leaf extract) and prepared NFCNDs in aqueous media. The absorption peak for *I. tinctoria* leaf extract appeared at 279 nm owing to the pi–pi* transition; it was obtained due to presence of polyphenols in the leaf extract and the aq. ammonia added to the reaction mixture, and then carbonization took place *via* condensation reactions to finally afford the NFCNDs.^41^

The two main characteristic peaks appeared for NFCNDs at 271 nm and 304 nm, which correspond to the pi–pi* transition for the sp2 carbon (C=C bonds) and n-pi* transitions of the carbonyl and amine functionalization on the NFCNDs surface, respectively.^42^ The UV-vis absorption values of the NFCNDs spectrum showed a red shift due to the introduction of ammonium functionalities by the addition of ammonia solution during the synthesis, and this could have efficiently converted the phenolic (–OH) groups into ammonium phenolate ions.^43^ The fluorescence excitation spectra of the NFCNDs (Fig. 2b) exhibited peaks at 310 nm, respectively, which evidently confirms the n–pi* transition. The UV-visible spectra of the synthesized NFCNDs indicated emission of cyan color (blue luminescence) under UV light at 365 nm.

**Fig. 3:**
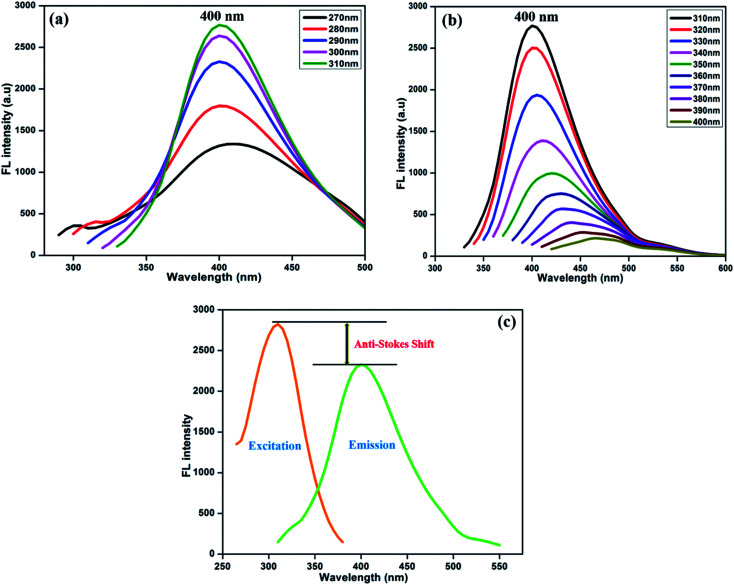
Fluorescence spectra of the prepared NFCNDs at different excitation wavelengths from (a) 270–310 nm and (b) 310–400 nm and (c) anti-Stokes shift of the NFCNDs.

Fig. 3 displays the fluorescence emission spectral responses of the synthesized NFCNDs at various excitation wavelengths. With increasing excitation wavelengths (*λ*_ex_) from 270 nm to 400 nm, the highest intensity of emission (*λ*_max_) was found at 400 nm. The fluorescence intensity (FL) was progressively increased by increasing *λ*_ex_ from 270 to 310 nm (Fig. 3a) owing to the pi–pi* transitions of the sp2 carbon cores. Furthermore (Fig. 3b), the emission intensity gradually decreased with increasing *λ*_ex_ from 310 nm to 400 nm, and the *λ*_max_ of 400 nm was observed at 310 nm. This specific fluorescence property of the NFCNDs may arise due to the anti-Stokes fluorescence (*λ*_max(ext)_ > *λ*_max(em)_), where two or more photons are equally absorbed by carbon particles, resulting in the emission of light.^44^ The fluorescence quantum yield (QY) for NFCNDs was found to be 12.6%. In our previous work, we had reported the preparation of self-passivated CDs using rice bran as a carbon source, and the obtained QY was 7.4%. It is confirmed that nitrogen is an effective functional moiety for the functionalization of CNDs, which would be helpful to alter their optical properties. For clear understanding, a comparison of the QYs and fluorescence nature of CDs prepared from different precursors with those of the synthesized NFCNDs is given in Table 1 (ESI).† Moreover, the highly fluorescent nature of the NFCNDs indicates that they are the most suitable probe for sensing, multi-color bio-imaging, and drug delivery applications.^45^

### 3.2 Morphology and size of the NFCNDs

**Fig. 4:**
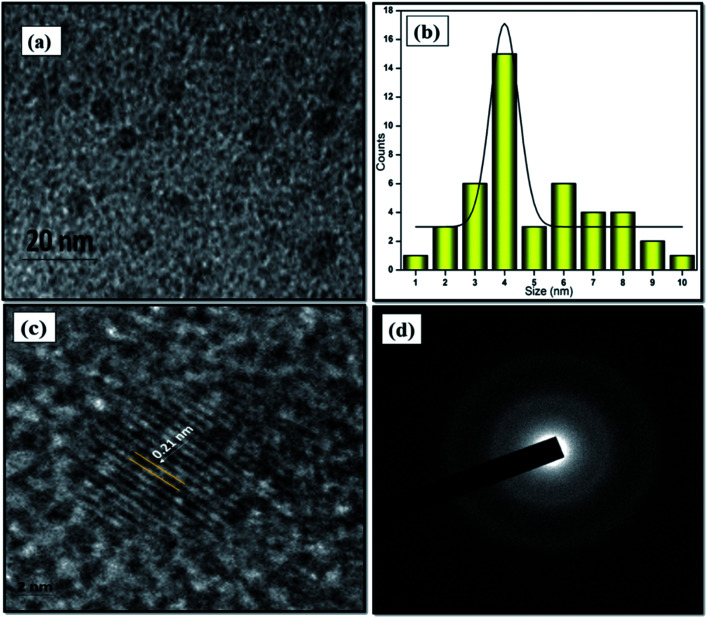
(a) TEM images of the formed NFCNDs at 20 nm, (b) histogram of the NFCNDs, (c) high-resolution image of the lattice fringes, (d) SAED outline of the NFCNDs.

The morphological structure and particle size of the formed NFCNDs were inspected by HR-TEM. Fig. 4a displays the spherical morphology of the formed NFCNDs, which clearly indicates that the carbon particles were evenly spread on the Cu grid. Fig. 4b shows the size distribution chart (ranging from 1.0 to 10 nm) and depicts that the formed NFCNDs have an average size of around 4 nm. The higher magnification TEM image (Fig. 4c) of the NFCNDs clearly displays the lattice fringes, and the calculated interlayer distance (*d*-spacing) is about 0.21 nm parallel to the (100) planes of graphite.^46^ Furthermore, Fig. 4d shows the selected area electron diffraction (SAED) outline of the NFCNDs, which confirmed the amorphous phase of carbon by the formation of clear circles.^33,34,40^

### 3.3 X-ray diffraction

**Fig. 5:**
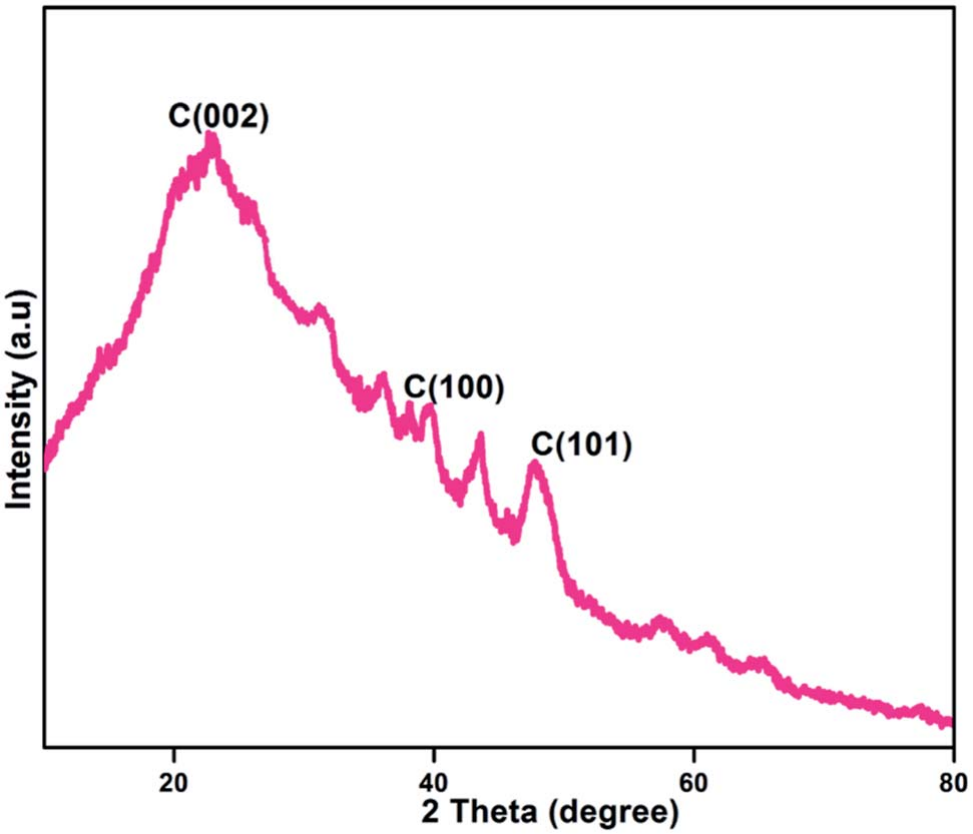
XRD spectrum of the NFCNDs.

Fig. 5 shows the X-ray diffraction (XRD) pattern of the formed NFCNDs. The XRD spectrum provides a wide peak at 2*θ* values of 22.73°, 43.6° and 47.7° for the C (002), C (100) and C (101) planes of graphitized carbon core, respectively.^47,48^ The amorphous phase of the NFCNDs is confirmed by the appearance of a broad peak at 2*θ* = 22.73°. Bragg's equation (*d* = *nλ*/2 sin *θ*) was used to calculate the *d*-spacing (interlayer distance) value for the formed NFCNDs. Using Bragg's equation, the *d*-spacing values were found to be 0.38 nm and 0.21 nm, which correspond to the C (002) and C (100) planes, respectively. These results could be associated with the calculated-spacing values from the HR-TEM lattice fringe images (Fig. 4(c)).^49^ These observations clearly confirmed that the formed NFCNDs generally have an amorphous structure due to the presence of well-defined graphitic carbon cores.

### 3.4 FTIR spectroscopy

**Fig. 6:**
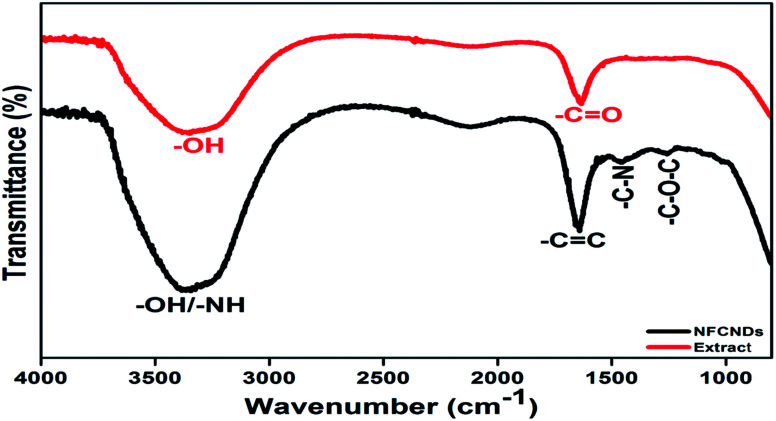
FTIR spectra of the *I. tinctoria* extract (red line) and NFCNDs (black line).

The FT-IR spectral responses of *I. tinctoria* extract and the formed NFCNDs are depicted in Fig. 6. Two absorption bands appeared in the IR spectrum of *I. tinctoria* leaf extract at 3336 and 1634 cm^−1^, which belong to the –OH str and –C=O str, respectively. The prepared NFCNDs showed absorption peaks at 3387, 1652, 1456, and 1261 cm^−1^, corresponding to the –OH/–NH, –C=C (sp2 carbon), –C–N (N functionalization), and –C–O–C functionalities; it was clearly observed that the functional moieties of *I. tinctoria* leaf extract were transferred due to the hydrothermal reaction, and the nitrogen was also functionalized in the NFCNDs.^50,51^ These FT-IR results revealed that acid functionalities, carbonyl groups, and amide, amine, and hydroxyl moieties were present in the aqueous soluble NFCNDs.

### 3.5 Raman spectra

**Fig. 7:**
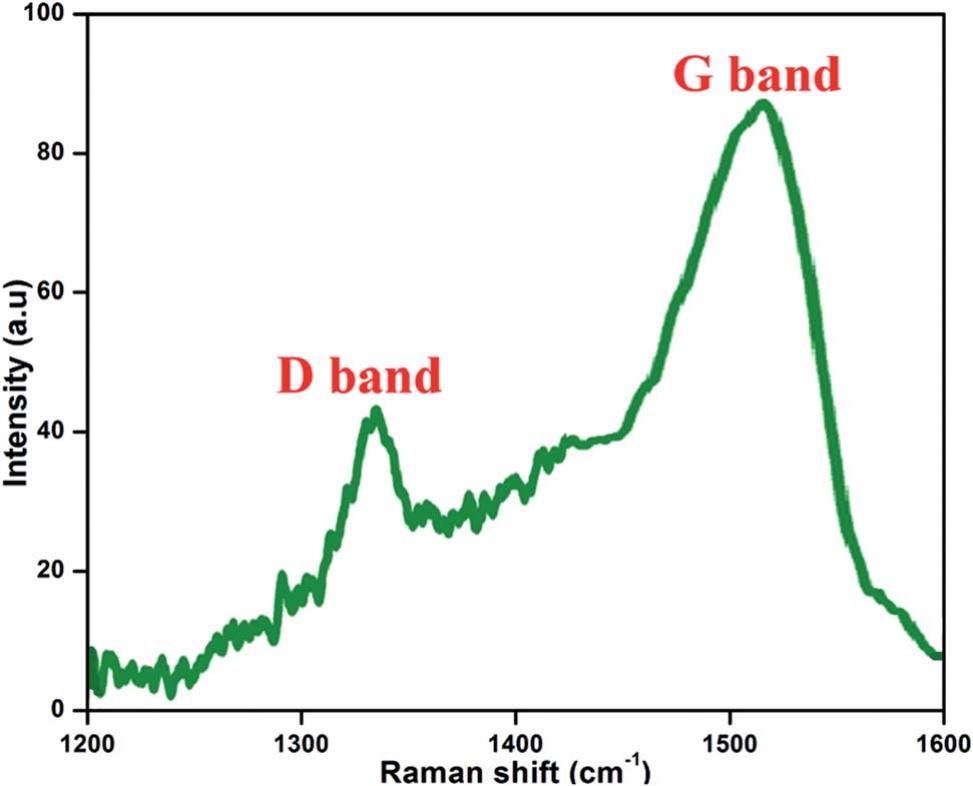
Raman spectra of the NFCNDs.

Fig. 7 presents the Raman spectrum of the prepared NFCNDs; this is the most valuable technique for the examination and confirmation of carbon-related nanomaterials. The Raman spectrum depicts two peaks at 1337 cm^−1^ and 1519 cm^−1^, which belong to the D band (defect region) and G band (graphitic region) in the NFCNDs, respectively. The D band arises due to sp3 defects, and the appearance of the G band indicates that the NFCNDs are mostly composed of sp2 carbon cores.^52^ The graphitic nature of the formed NFCNDs was determined by the degree of graphitization. The intensity ratio between the D and G bands (*I*_D_/*I*_G_) is less than 1 for ideal graphite.^53^ Here, the *I*_D_/*I*_G_ value was found to be 0.49, which depicts the moderate graphite structure of the prepared NFCNDs and also confirms the existence of minimal surface defects in the graphitic structure of the NFCNDs.

### 3.6 Thermal stability of the NFCNDs

**Fig. 8:**
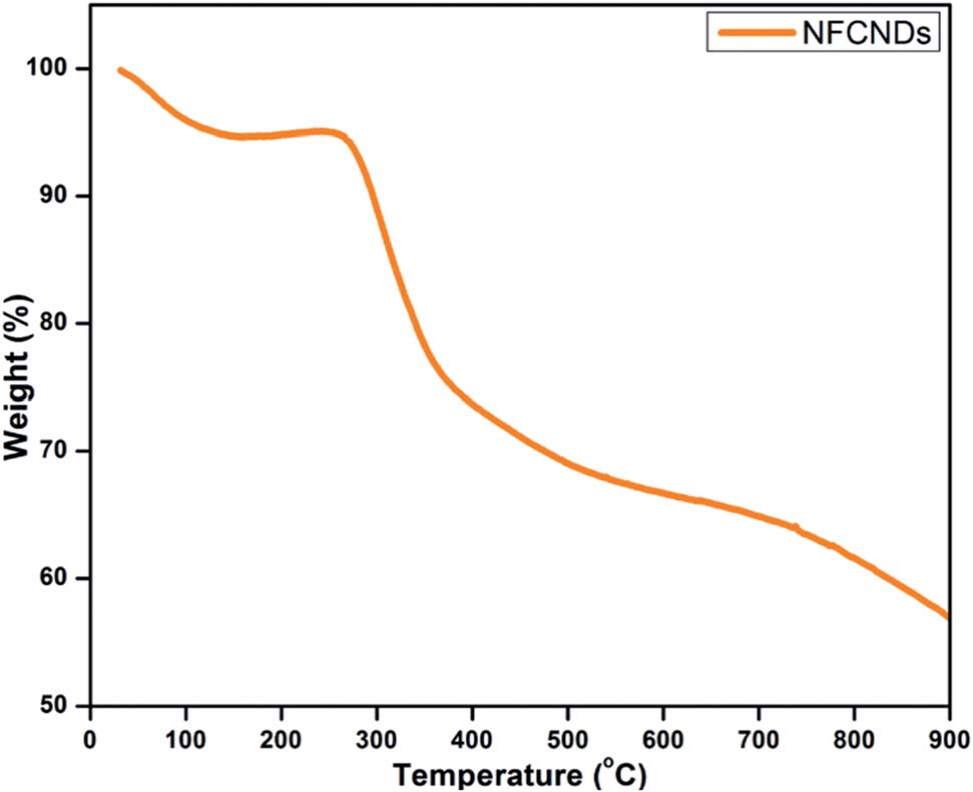
TGA plot of the NFCNDs.

Thermogravimetric analysis (TGA) was employed to determine the thermal stability of the synthesized NFCNDs, as shown in Fig. 8. In the beginning, a 12% weight loss occurred owing to the decomposition of the water molecules. Further, a consequent weight loss was acquired from 300–400 °C, which was ascribed to the desorption of the hydroxyl and carboxyl groups. When the temperature was raised to 700 °C, a 60% weight loss was observed, which could be due to the elimination of functional groups on the carbon skeleton of the prepared NFCNDs.^54^

### 3.7 X-ray photoelectron spectroscopy

**Fig. 9:**
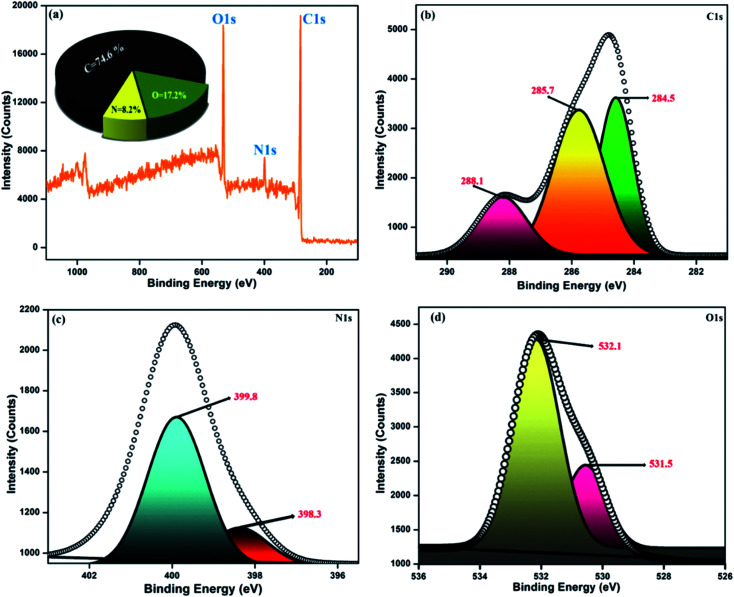
(a) Full survey spectrum of the synthesized NFCNDs (inset: elemental percentage graph); (b) C 1s, (c) N 1s, (d) O 1s.

The diverse surface electronic states and elemental composition of the NFCNDs were explored by X-ray photoelectron spectroscopy (XPS). Fig. 9a depicts the survey spectrum of the NFCNDs, which displayed the C 1s (carbon), N 1s (nitrogen), and O 1s (oxygen) peaks at 285, 399, and 530 eV, respectively. The C 1s spectra were deconvoluted into three distinct peaks at 284.5, 285.7, and 288.1 eV, as shown in Fig. 9b, which were ascribed to C–H, C=C (sp2), and C–N bonds, respectively.^52^Fig. 9c demonstrates the deconvoluted N 1s spectra, which show two diverse peaks at 398.3 and 399.8 eV owing to the amine (C–NH_2_) and amide (O=C–NH_2_) groups existing in the formed NFCNDs. The high resolution (O 1s) spectrum (Fig. 9d) provides two disparate peaks at 531.5 and 532.1 eV, which can be attributed to the C=O and C–OH/C–O–C functionalities in the NFCNDs, respectively.^55^ Finally, the XPS spectrum suggests that the atomic percentages of the elements in the NFCNDs were C (74.6%), N (8.2%), and O (17.2%). From the overall discussion, it is evident that amine, hydroxyl, carboxyl and amide functional groups are present in the formed NFCNDs.

## 4 Applications of the NFCNDs

### 4.1 Photocatalytic activity of the NFCNDs

**Fig. 10:**
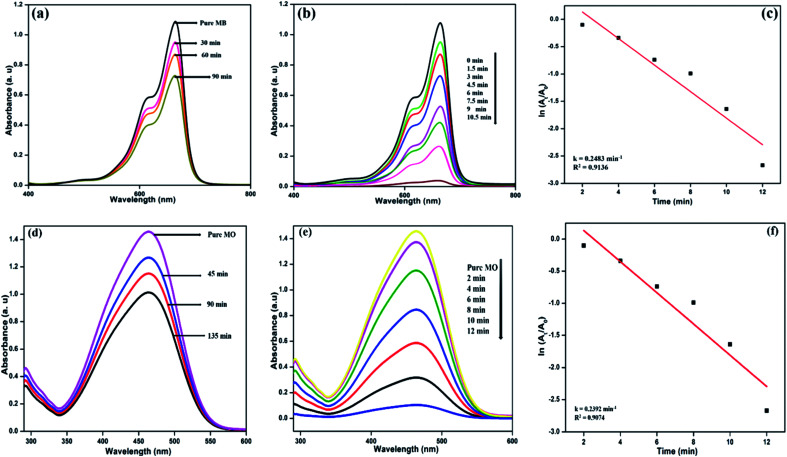
UV-vis spectra of the MB/MO reduction using sodium borohydride (a and d) without NFCNDs and (b and e) with NFCNDs; (c and f) pseudo first order kinetics plots for the reduction of MB/MO with NFCNDs.

The photocatalytic activity of the formed NFCNDs was evaluated through catalytic reduction of MB and MO dyes. The absorption intensity of blue MB dye was at 664 nm and that of orange MO was at 466 nm. The prepared NFCNDs served as a green catalyst in this environmental remediation process. Photocatalytic reduction of MB and MO dyes performed under light irradiation with an ice-cooled conditioned reducing agent (NaBH_4_) and in the presence and absence of NFCNDs (green catalyst) was investigated in this work. Fig. 10a shows the UV-vis absorption spectra of MB and MO after UV light irradiation using NaBH_4_ without prepared NFCNDs for a regular period. The intensity of MB and MO (Fig. 10a and d) was found to decrease to the minimum extent after 90 min and 135 min, respectively, which displays no significant change in intensity; this indicates that complete reduction does not occur. On the other hand, when the prepared NFCNDs (60 microliters) were added to the mixtures of MB and MO containing sodium borohydride, a significant reduction was observed in the intensity of the dyes (Fig. 10b and e). Finally, the reduction of MB was completed within 10.5 min, and that of MO was completed in 12 min by addition of the efficient green catalyst NFCNDs. Further, the photocatalytic degradation of MB and MO dyes was investigated using undoped CNDs, and the results are given in ESI Fig. 1.† Even after 120 min, no sigficant reduction was attained while using undoped CDs as a catalyst. In the case of using NFCNDs as a catalyst, the photocatalytic degradation of MB and MO was completed in a very short period of time compared to the undoped CNDs catalyst. The results confirmed that the catalytic activity of the NFCNDs effectively improved the rate of the reduction of the MB and MO dyes using NaBH_4_. This could be achieved by decreasing the activation energy of the reduction reaction owing to the incorporation of NFCNDs into the reduction process.^56^

The determination of the rate of the reaction (*K*_cat_) for the dye reduction of MB/MO by the prepared NFCNDs (catalyst) could be calculated from the pseudo-first-order kinetics. Fig. 10c and f show the pseudo-first-order kinetics plots for the reduction of MB and MO using NFCNDs. The rate constants (*K*_cat_) and coefficients (*R*^2^) could be obtained from the slopes of the linear plots; the *K*_cat_ and *R*^2^ values of MB and MO reduction were found to be 0.2483 min^−1^ and 0.9136 and 0.2392 min^−1^ and 0.9074, respectively. The acquired *K*_cat_ values for the reduction of MB and MO by the NFCNDs were higher than those in earlier reports.

This photocatalytic catalytic reduction of MB and MO could be explored using the well-known Langmuir Hinshelwood mechanism.^56^ The MB and MO dyes function as electrophiles, and the borohydride (BH_4_^−^) ions act as a nucleophile. Concurrent adsorption takes place above the surface of the NFCNDs, and the transformation of electrons occurs from BH_4_^−^ ions to MB and MO through the green catalyst. The synthesized NFCNDs act as an electron transfer mediator between dye molecules and BH_4_^−^ ions. This only leads to the reduction reaction of MB and MO dyes. During this reaction, electrons from BH_4_^−^ ions transfer from the VB to the CB in the NFCNDs. Due to this electron transfer, a hole is created in the VB, and this hole reacts with H_2_O and O_2_ to produce hydroxide (OH˙) and oxygen (O_2_˙) radicals, simultaneously. If only O_2_˙ radicals are formed, the reduction reaction takes place at a comparatively slow rate. The formation of both radicals led to the reduction reactions of MB and MO, which were thus completed in a short period. NFCNDs efficiently served as a mediator for these electron transformations, and this suggests reduction of the energy obstacles among the starting materials (reactants) and final products.^57^ Hence, the wide surface to volume ratio and effective electron withdrawing potential of the synthesized NFCNDs are the major reasons for this remarkable catalytic photocatalytic reduction.^40^

**Fig. 11:**
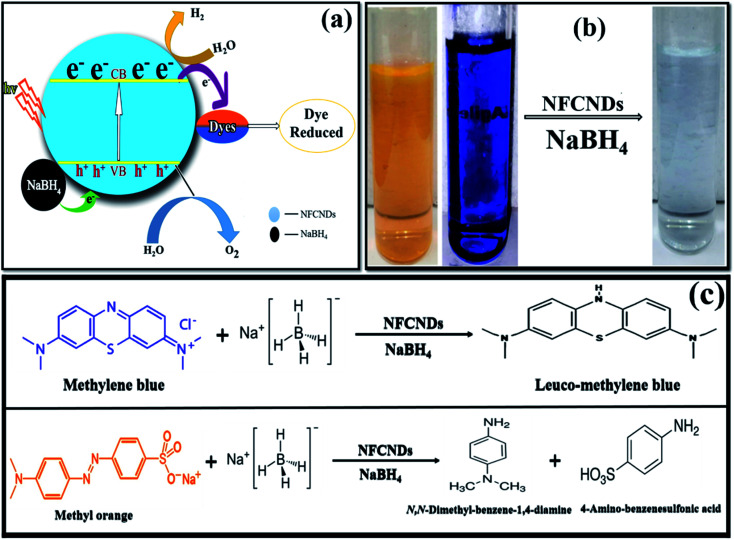
(a) Possible reduction mechanism. (b) Photographs of the initial dyes and treated water. (c) Reaction reduction mechanism of MB/MO.

Fig. 11a displays the possible mechanisms of the reduction of MB and MO. Finally, photographs of the decolorized dye solutions are shown in Fig. 11b, and schematics of the reduction reactions of the dyes are also shown in Fig. 11c. The toxic MB and MO dyes were converted into eco-friendly products through the green catalyst. The results conclude that the prepared NFCNDs were proved to be an effective alternate for metal nanocatalysts in the removal of environmental pollution.^58^

### 4.2 NFCNDs as a fluorescent ink

**Fig. 12:**
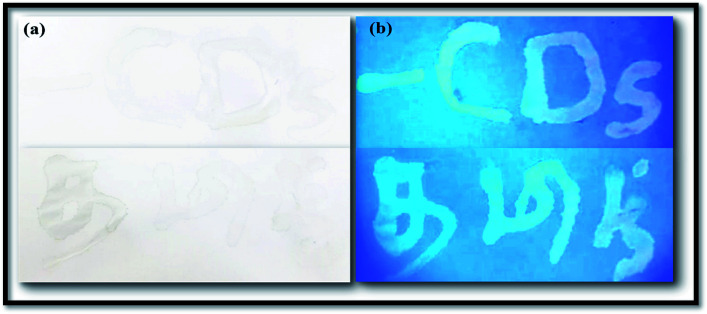
Images of fluorescent ink-written paper: (a) naked eye view and (b) under UV light (365 nm).

Finding an innovative application which is suitable for developing materials such CNDs is economically important. Currently, a wide range of the dyes, coloring agents, and polymers that are used for various industrial and forensic applications are extremely toxic and also have high cost. Here, the synthesized NFCNDs possess unique properties, high biocompatibility, strong fluorescence, a low/nontoxic nature, and transparency in the visible region, which encourages the use of this carbon nanomaterial as an alternate candidate for various important applications. In this recent scenario, the solubilized fluorescent NFCNDs were utilized as a fluorescent ink in anti-counterfeiting applications. The prepared fluorescent ink containing NFCNDs was filled in an empty ink pen; some letters were written on white paper, allowed to dry at room temperature and placed under UV light (365 nm). Fig. 12a displays the image of the white paper in naked eye view, and no visible letters can be observed; however, Fig. 12b depicts the image of the same white paper under 365 nm UV light, which shows clearly visible blue fluorescent patterns of the written letters when subjected to ultra-violet light irradiation, and these letters can be easily removed by water. The visible patterns evidently indicate the cyan fluorescence emission of the NFCNDs and are also helpful to extend the applications of the material to the forensic and anti-counterfeiting fields.

## 5 Conclusion

Here, we reported the synthesis of highly fluorescent nitrogen functionalized CNDs using *Indigofera tinctoria* leaf extract as a novel carbon source and aq. ammonia as a nitrogen source by a cost-effective, uncomplicated, and eco-friendly single-step hydrothermal process. The obtained NFCNDs exhibits good water solubility, greater QY, and strong fluorescence. The optical properties were studied by UV-visible and fluorescence spectrometry. The size of the synthesized NFCNDs was 4 nm in diameter, and a spherical morphology was found from the HR-TEM studies. The XRD results revealed that the amorphous character of the NFCNDs as well as the FT-IR and XPS studies evidently confirmed the functionalization of nitrogen on the CNDs surface. From the environmental point of view, the study describes the catalytic efficiency of NFCNDs explored for the reduction of methylene blue and methyl orange dyes by using NaBH_4_. Complete reduction was attained within 10.5 min for MB and 12 min for MO using the NFCNDs green catalyst. Moreover, the NFCNDs were utilized as a biocompatible fluorescent ink and also emitted cyan color under UV light. Overall, the study enlightened the uses of a novel green catalyst for environmental remediation and also identified a promising probe for anti-counterfeiting applications.

## Data availability

All data generated or analyzed during this study are included in this published article.

## Author contributions

Jothi Vinoth Kumar: conceptualization, methodology, writing – original draft. Ganesan Kavitha: data curation, formal analysis. Rajaram Arulmozhi: visualization. Velusamy Arul: data curation. Natarajan Abirami: supervision, investigation.

## Conflicts of interest

Authors declared that there are no conflicts of interest.

## Supplementary Material

RA-011-D1RA04351J-s001
