# Supplementary material for: Cyan color-emitting nitrogen-functionalized carbon nanodots (NFCNDs) from Indigofera tinctoria and their catalytic reduction of organic dyes and fluorescent ink applications
Source: RSC Adv. 2021 Aug 16;11(44):27745–56. doi: 10.1039/d1ra04351j (PMC9037855; doi:10.1039/d1ra04351j)
Supplement: RA-011-D1RA04351J-s001 [file RA-011-D1RA04351J-s001.pdf]

Electronic Supplementary Material (ESI) for RSC Advances.
This journal is © The Royal Society of Chemistry 2021

Electronic Supplementary Material (ESM)

Cyan color -emitting Nitrogen functionalized carbon nanodots (NFCNDs) from *Indigofera tinctoria* and their catalytic reduction of organic dyes and fluorescent ink applications

Jothi VinothKumar[a], Ganesan Kavitha[a], Rajaram Arulmozhi[a], Velusamy Arul[b], and Natarajan Abirami[a*]

[a]Department of Chemistry, Faculty of Engineering and Technology, SRM Institute of Science and Technology, Kattankulathur, Tamil Nadu-603 203, India.

[b] Department of Science and Humanities, Sri Venkateswaraa College of Technology, Vadkkal, Sriperumbudur, Chennai-602 105, Tamil Nadu, India.

*Corresponding Author: E-mail: abiramin@srmist.edu.in (Dr. N. Abirami)

Table 1. Comparison table on QY and fluorescence nature of NFCNDs with previous reports

Precursor	Types of CDs	QY (%)	Fluorescence	Ref.
Tamarind	FL- CDs	6.48	Green	1
Rice Bran	Pure CDs	7.4	Green	2
Apple	Pure CDs	4.27	Blue	3
Streptomycin	N-CDs	7.6	Blue	4
Carrot juice	Pure CDs	5.16	Blue	5
Gynostemma	Pure CDs	5.7	Green	6
Red cabbage	Pure CDs	8.3	Blue	7
Zingiberis rhizoma	Pure CDs	5.2	Green	8
White pepper	Pure CDs	10.4	Green	9
Indigofera tinctoria	N-CDs	12.6	Cyan blue	Present work

Note: FL- Fluorescence; CDs- Carbon dots; N-CDs – Nitrogen Carbon dots.

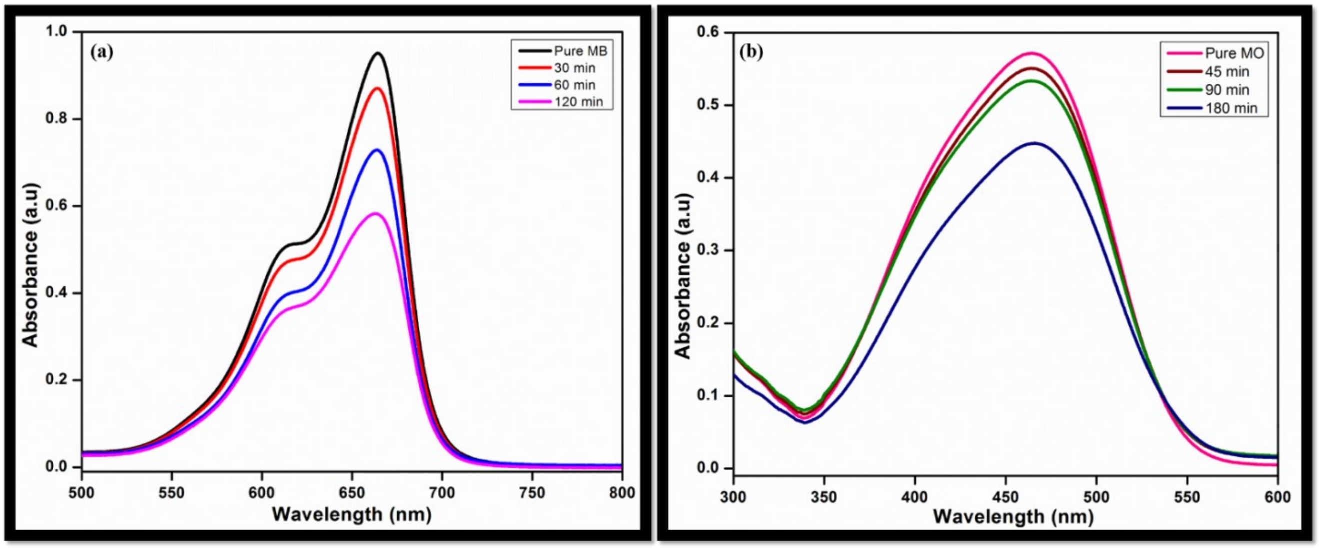

Figure ESM 1. (a) Undoped CNDs for MB (b) Undoped CNDs for MO

References

1 M. A. Jhonsi and S. Thulasi, *Chem. Phys. Lett.*, 2016, **661**, 179–184.

2 V. K. Jothi, K. Ganesan, A. Natarajan and A. Rajaram, *J. Fluoresc.*, 2021, **31**, 427–436.

3 V. N. Mehta, S. Jha, H. Basu, R. K. Singhal and S. K. Kailasa, *Sens. Actuators, B*, 2015, **213**, 434–443.

4 J. Zhou, Z. Sheng, H. Han, M. Zou and C. Li, *Mater. Lett.*, 2012, **66**, 222–224.

5 Y. Liu, Y. Liu, M. Park, S. J. Park, Y. Zhang, M. R. Akanda, B. Y. Park and H. Y. Kim, *Carbon Letters*, 2017, **21**, 61–67.

6 X. Wei, L. Li, J. Liu, L. Yu, H. Li, F. Cheng, X. Yi, J. He and B. Li, *ACS Appl. Mater. Interfaces*, 2019, **11**, 9832–9840.

7 N. Sharma, G. S. Das and K. Yun, *Appl. Microbiol. Biotechnol.*, 2020, **104**, 7187–7200.

8 M. Zhang, J. Cheng, Y. Zhang, H. Kong, S. Wang, J. Luo, H. Qu and Y. Zhao, *Nanomedicine*, 2020, **15**, 851–869.

9 R. Long, Y. Guo, L. Xie, S. Shi, J. Xu, C. Tong, Q. Lin and T. Li, *Food Chem.*, 2020, **315**, 126171.
